# Throughput Analysis of Buffer-Aided Decode-and-Forward Wireless Relaying with RF Energy Harvesting

**DOI:** 10.3390/s20041222

**Published:** 2020-02-23

**Authors:** Phat Huynh, Khoa T. Phan, Bo Liu, Robert Ross

**Affiliations:** 1 Department of Engineering, La Trobe University, Bundoora 3086, Australia; R.Ross@latrobe.edu.au; 2 Department of Computer Science and Information Technology, La Trobe University, Bundoora 3086, Australia; K.Phan@latrobe.edu.au; 3 School of Computer Science, University of Technology Sydney, Ultimo 2007, Australia; Bo.Liu@uts.edu.au

**Keywords:** vehicular communication, green communication, energy harvesting, buffer-aided relaying

## Abstract

In this paper, we investigated a buffer-aided decode-and-forward (DF) wireless relaying system over fading channels, where the source and relay harvest radio-frequency (RF) energy from a power station for data transmissions. We derived exact expressions for end-to-end throughput considering half-duplex (HD) and full-duplex (FD) relaying schemes. The numerical results illustrate the throughput and energy efficiencies of the relaying schemes under different self-interference (SI) cancellation levels and relay deployment locations. It was demonstrated that throughput-optimal relaying is not necessarily energy efficiency-optimal. The results provide guidance on optimal relaying network deployment and operation under different performance criteria.

## 1. Introduction

Internet of things (IoT) is a major technology of the incoming industrial revolution 4.0 [1]. An IoT network consist of a large number of connected devices, each of which requires a reliable energy supply for efficient operation [2]. This requirement can be satisfied by embedding batteries into the IoT devices, possibly incurring high-cost and safety-related issues to replace [3]. Harvesting energy from the ambient environment, such as solar, wind, thermal and radio frequency (RF) signals to empower electronic devices is becoming the future of IoT [4]. In particular, energy harvesting (EH) from RF signals has attracted significant interest because it can offer simultaneous wireless information and power transfer (SWIPT) while other natural energy sources are usually reliant on the climate of deployment locations [5,6,7]. Such SWIPT-based systems are inherently useful in applications with hard-to-access devices such as inside bodies, building structures, vehicles, or remote areas [8]. In particular, EH-based communications systems are suitable for intelligent transportation systems (ITS) [9,10,11], where vehicles form a vehicle-to-vehicle (V2V) wireless communication network. This V2V communication can take place in transit with moving transmitters and receivers, as well as in slow-changing environments such as car parks, where the devices are more static. Moreover, full-duplex communication is a mature field in wireless communication. There have been numerous works in the literature on the combination of energy harvesting and full-duplex for more advanced and self-sustain communication [12,13].

In this paper, we studied a buffer-aided dual-hop wireless relaying communication model, where the source and relay harvest RF energy from a dedicated power station for data transmission to the destination. Our model represents a simple yet powerful EH-based system where all the communication devices are energy self-sufficient by harvesting RF energy. Along this line of research, existing literature has proposed and analyzed several wireless relaying models using RF energy harvesting. For example, in [5] and [6], the relay harvests RF energy from the source, which has access to a reliable power source. The work [7] alternatively considers that the source harvests energy from the relay. The authors investigated two cases: without battery storage and with unlimited battery capacity. In contrast, our work has both the source and the relay as EH-based nodes. While such an assumption has been considered in [14,15], our work considers a data buffer at the relay for (end-to-end) throughput enhancement with both half-duplex (HD) and full-duplex (FD) relaying modes. In [16], the authors developed an efficient wireless energy transfer (WET) policy for a multiple-node communication network that is powered by a single energy access point (E-AP). This work focuses on the resource allocation for a network where communications between nodes are direct transmissions. In reality, direct transmissions often encounter challenges in extended distances due to hostility on the channel. Our paper considers a wireless buffer-aided relaying communication scheme that is particularly useful to ensure quality-of-service (QoS) in a long transmission range. Overall, the main contributions of our work can be summarized as follows:

(1) We derive analytical throughput expressions in buffer-aided decode-and-forward HD and FD relaying modes. The expressions also take into account the fading channel statistics, EH and data transmission duration, as well as the self-interference (SI) cancellation level (for FD relaying). These expressions can be exploited to determine the parameters for optimal throughput performance.

(2) In addition to the throughput, we also investigate the energy efficiency of the relaying schemes. This performance benchmark is particularly relevant for green V2V communication applications.

(3) We perform numerical simulations to demonstrate the throughput and energy efficiency performance of the relaying schemes under different SI cancellation levels and relay locations. The results showed that with sufficiently small residual SI, FD relaying achieves higher throughput and energy efficiency than HD relaying. Moreover, it is revealed that throughput-optimal relaying is not necessarily energy efficiency-optimal in general. The results provide guidance on optimal relaying network deployment and operation under different performance criteria.

## 2. System Model and Throughput Analysis

### 2.1. System Model

**Figure 1:**
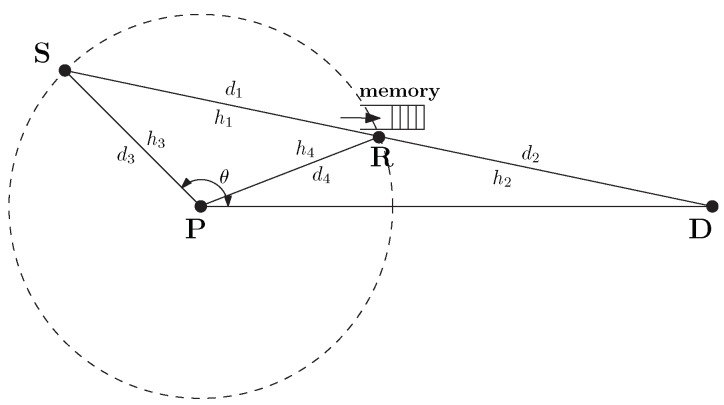
A wireless communication system consists of energy-constrained source and relay.

**Figure 2:**
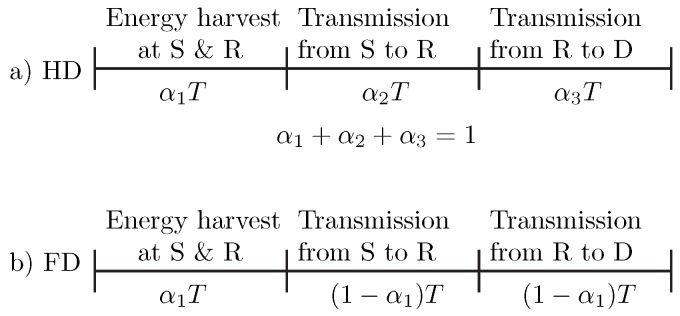
Time allocation in a transmission block: (**a**) Half duplex, (**b**) Full duplex.

The source node, **S** and the relay, **R** harvest energy from the power station, **P** for data transmission from **S** to **D** (Figure 1). We assume that **R** only harvest energy from **P** and does not perform self-energy recycling from its own transmission energy. The complex channel coefficients on the **SR**, **RD**, **PS** and **PR** channels are denoted by $h_{1}$, $h_{2}$, $h_{3}$ and $h_{4}$ respectively. $d_{1}$, $d_{2}$, $d_{3}$ and $d_{4}$ denote the distances of the communication channels corresponding with their channel coefficient notations. The angle between the **SP** and **PD** channels is denoted by θ. Figure 2 illustrates the time allocations in a transmission block in HD and FD relaying modes. The block time *T* is the total time for EH and information transmissions from **S** to **D** via the relay. For simplicity, it is assumed that the fading-block duration equals to *T* within which the channel coefficients remain constant and vary independently over fading blocks. $\alpha_{1}$ indicates the harvesting time. In the HD mode, the data transmission time is divided into two parts $\alpha_{2}$ and $\alpha_{3}$ for information transmission time on **SR** and **RD** channels, respectively. In contrast, FD communication features simultaneous data reception and transmission at the relay; hence, the time allocated for these two transmissions is simply $1-\alpha_{1}$.

**Figure 3:**
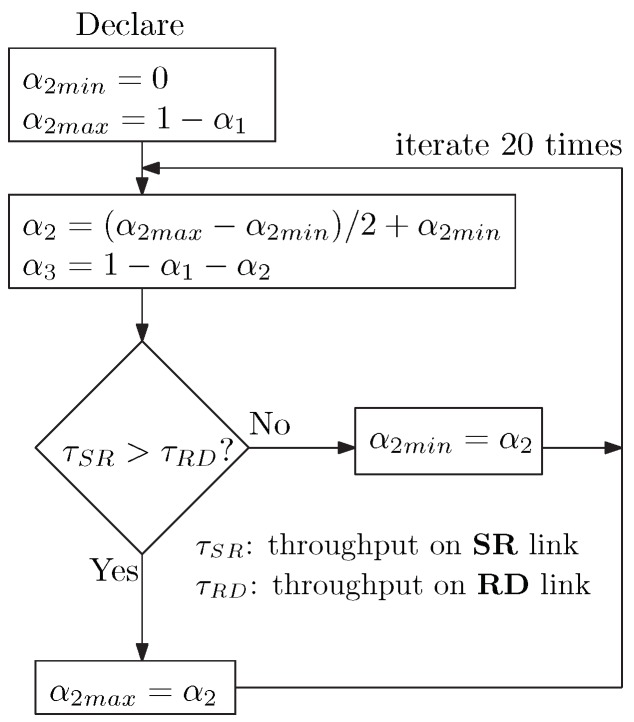
Process to determine optimal $\alpha_{2}$ and $\alpha_{3}$ values.

We can see that for a given $\alpha_{1}$, when $\alpha_{2}$ increases, the throughput on the **SR** channel increases, but it suppresses the throughput on the **RD** channels, and vice versa. Hence, optimal values of $\alpha_{2}$ and $\alpha_{3}$ respectively equalize the throughput on the **SR** and **RD** channels. The process to determine optimal $\alpha_{2}$ and $\alpha_{3}$ is illustrated in Figure 3.

The energy harvested at **S** and **R** is given by:

(1) $$E_{s}=\eta P|h_{3}{|}^{2}\alpha_{1}T,\;E_{r}=\eta P{\left|h_{4}\right|}^{2}\alpha_{1}T$$

where 0<η<1 is the energy conversion efficiency of energy harvesting circuitry at **S** and **R**, *P* is the RF signal power broadcast by the power source. The channel power gains are computed using the standard path loss model:

(2) $$|h_{p}{|}^{2}={\left(\frac{c}{4\pi f_{c}}\right)}^{2}d_{p}^{-m}e_{p}=Fd_{p}^{-m}e_{p},\;\;p\in \left\{1,2,3,4\right\}$$

where $F≜{\left(\frac{c}{4\pi f_{c}}\right)}^{2}$, *c* is the speed of light, $f_{c}$ is the carrier frequency, *m* is the path loss exponent. Rayleigh fading channels are assumed because we consider a general deployment in reality where the channels between the power station and the devices are multi-paths. As a result, $e_{p}$ is a complex exponential random variable with unit mean.

### 2.2. Half-Duplex (HD) Relaying

We first consider HD relaying. The transmission powers $P_{s,HD}$ of **S** and $P_{r,HD}$ of **R** are given by:

(3) $$P_{s,HD}=\frac{E_{s}}{\alpha_{2}T}=\frac{\eta P|h_{3}{|}^{2}\alpha_{1}}{\alpha_{2}},\;P_{r,HD}=\frac{E_{r}}{\alpha_{3}T}=\frac{\eta P|h_{4}{|}^{2}\alpha_{1}}{\alpha_{3}}.$$

The signal-to-noise ratios (SNR) at the relay, $\gamma_{r,HD}$, and at the destination, $\gamma_{d,HD}$ are given by:

(4) $$\gamma_{r,HD}=\frac{\eta P|h_{1}{|}^{2}{\left|h_{3}\right|}^{2}\alpha_{1}}{\alpha_{2}\sigma_{sr}^{2}},\;\gamma_{d,HD}=\frac{\eta P|h_{2}{|}^{2}{\left|h_{4}\right|}^{2}\alpha_{1}}{\alpha_{3}\sigma_{rd}^{2}}.$$

where $\sigma_{sr}^{2}$ and $\sigma_{rd}^{2}$ are variances of the AWGN noise at the relay and destination, respectively.

With a buffer-aided relay system, the (end-to-end) throughput $\tau_{HD}$ is given by [17]:

(5) $$\begin{array}{cc} \tau_{HD} & =\min\{E\left[\alpha_{2}\log_{2}\left(1+\gamma_{r,HD}\right)\right],E\left[\alpha_{3}\log_{2}\left(1+\gamma_{d,HD}\right)\right]\}\\ \; & =\min\{\alpha_{2}C_{r,HD},\alpha_{3}C_{d,HD}\} \end{array}$$

where $C_{r,HD}$ and $C_{d,HD}$ are the ergodic capacities of the **SR** and the **RD** channels, respectively. E[.] denotes the statistical expectation over fading channels. We also assumed the timescales of energy harvesting and channel fading block duration is sufficiently long so that long codeword transmissions are possible to achieve the capacity. In the case of short packets (or finite blocklength code) [18], only smaller rates than the capacity log(1 + SNR) are achieved and hence, the throughput obtained in our work will serve as the upper bounds. If the system were using non-buffer relaying mode, the throughput would be given by:

(6) $$E[\min\left\{\alpha_{2}\log_{2}\left(1+\gamma_{r,HD}\right),\alpha_{3}\log_{2}\left(1+\gamma_{r,HD}\right)\right\}].$$

Mathematically, we can see that the throughput of a buffer-aid system is always higher than a non-buffer one [17].

In order to find the analytical expression for $C_{r,HD}$, we firstly evaluate the cummulative distribution function (CDF) of $\gamma_{r,HD}$, $F_{\gamma_{r,HD}}\left(\gamma \right)$ and then evaluate the probability distribution function (PDF) of $\gamma_{r,HD}$, $f_{\gamma_{r,HD}}\left(\gamma \right)$. The CDF $F_{\gamma_{r,HD}}\left(\gamma \right)$ is given by:

(7) $$F_{\gamma_{r,HD}}=\mathrm{Pr}\left(\gamma_{r,HD}<\gamma \right)=1-t_{1}K_{1}\left(t_{1}\right)$$

where Pr(.) denotes probability operator, $K_{1}\left(.\right)$ is the first-order of the second kind modified Bessel function, $t_{1}=\sqrt{\frac{4b_{1}\gamma }{\lambda_{1}\lambda_{3}}}$, $b_{1}=\frac{\alpha_{2}\sigma_{sr}^{2}}{\eta P\alpha_{1}F^{2}d_{1}^{-m}d_{2}^{-m}}$ and $\lambda_{1}$ and $\lambda_{3}$ are the mean values of the exponential random variables $|h_{1}{|}^{2}$ and $|h_{3}{|}^{2}$, respectively.

**Proof.**  Appendix A

The PDF $f_{\gamma_{r,HD}}\left(\gamma \right)$ is then given by (using [19], 8.486.18):

(8) $$\begin{array}{cc} f_{\gamma_{r,HD}}\left(\gamma \right) & =\frac{\partial (F_{\gamma_{r,HD}})}{\partial \gamma }\\ \; & =t_{1}K_{0}\left(t_{1}\right)t_{1}^{\prime }\;\\ \; & =\frac{2b_{1}}{\lambda_{1}\lambda_{3}}K_{0}\left(t_{1}\right). \end{array}$$

The capacity of the **SR** channel, $C_{r,HD}$ is:

(9) $$\begin{array}{cc} C_{r,HD} & =\int_{0}^{\infty }f_{\gamma_{r,HD}}\left(\gamma \right)\log_{2}\left(1+\gamma \right)d\gamma \\ \; & =\int_{0}^{\infty }\frac{2b_{1}}{\lambda_{1}\lambda_{3}}K_{0}\left(t_{1}\right)\log_{2}\left(1+\gamma \right)d\gamma . \end{array}$$

Similarly, the capacity of the **RD** channel, $C_{d,HD}$ can be implied as:

(10) $$C_{d,HD}=\int_{0}^{\infty }\frac{2b_{2}}{\lambda_{2}\lambda_{4}}K_{0}\left(t_{2}\right)\log_{2}\left(1+\gamma \right)d\gamma$$

where $t_{2}=\sqrt{\frac{4b_{2}\gamma }{\lambda_{2}\lambda_{4}}}$. $b_{2}=\frac{\alpha_{3}\sigma_{rd}^{2}}{\eta P\alpha_{1}F^{2}d_{2}^{-m}d_{4}^{-m}}$ and $\lambda_{2}$ and $\lambda_{4}$ are the mean values of the exponential random variables $|h_{2}{|}^{2}$ and $|h_{4}{|}^{2}$, respectively. ☐

### 2.3. Full-Duplex (FD) Relaying

We now consider FD relaying. The transmission powers $P_{s,FD}$ and $P_{r,FD}$ in FD communication, are given by:

(11) $$P_{s,FD}=\frac{E_{s}}{(1-\alpha_{1})T},\;P_{r,FD}=\frac{E_{r}}{(1-\alpha_{1})T}.$$

Under FD relaying, data reception at the relay suffers SI generated by the its own transmission signal, in addition to the AWGN noise.

The capacity of the **RD** channel, $C_{d,FD}$ is analogous to the case of HD relaying:

(12) $$C_{d,FD}=\int_{0}^{\infty }\frac{2b_{3}}{\lambda_{2}\lambda_{4}}K_{0}\left(t_{3}\right)\log_{2}\left(1+\gamma \right)d\gamma$$

where $t_{3}=\sqrt{\frac{4b_{3}\gamma }{\lambda_{2}\lambda_{4}}}$ and $b_{3}=\frac{\left(1-\alpha_{1}\right)\sigma_{rd}^{2}}{\eta P\alpha_{1}F^{2}d_{2}^{-m}d_{4}^{-m}}$. On the other hand, the SNR of the **SR** link, $\gamma_{r,FD}$, is given by:

(13) $$\gamma_{r,FD}=\frac{P_{s,FD}{\left|h_{1}\right|}^{2}}{\beta P_{r,FD}+\sigma_{sr}^{2}}=\frac{\eta P|h_{1}{|}^{2}{\left|h_{3}\right|}^{2}\alpha_{1}}{\eta P\beta |h_{4}{|}^{2}\alpha_{1}+\left(1-\alpha_{1}\right)\sigma_{sr}^{2}}$$

where β is the residual SI noise factor. The CDF of $\gamma_{r,FD}$, $F_{\gamma_{r,FD}}\left(\gamma \right)$ is given by:

(14) $$F_{\gamma_{r,FD}}\left(\gamma \right)=1-\frac{1}{\lambda_{4}}\int_{0}^{\infty }e^{-z/\lambda_{4}}t_{4}K_{1}\left(t_{4}\right)dz$$

where $t_{4}=\sqrt{\frac{4\gamma (b_{4}z+c_{4})}{a_{4}\lambda_{1}\lambda_{3}}}$, $a_{4}=\eta P\alpha_{1}F^{2}d_{1}^{-m}d_{3}^{-m}$, $c_{4}=\left(1-\alpha_{1}\right)\sigma_{sr}^{2}$ and $b_{4}=\eta P\beta \alpha_{1}Fd_{4}^{-m}$.

**Proof.**  Appendix B

The PDF of $\gamma_{r,FD}$, $f_{\gamma_{r,FD}}\left(\gamma \right)$ is then given by:

(15) $$\begin{array}{cc} f_{\gamma_{r,FD}}\left(\gamma \right) & =\frac{\partial F_{\gamma_{r,FD}}\left(\gamma \right)}{\partial \gamma }\\ \; & =\frac{1}{\lambda_{4}}\int_{0}^{\infty }e^{-\frac{z}{\lambda_{4}}}t_{4}K_{0}\left(t_{4}\right)t_{4}^{\prime }dz\;(\mathrm{Using}\;\mathrm{Leibniz}’s\;\mathrm{rule})\\ \; & =\frac{2}{\lambda_{1}\lambda_{3}\lambda_{4}}\int_{0}^{\infty }e^{-\frac{z}{\lambda_{4}}}K_{0}\left(t_{4}\right)\frac{b_{4}z+c_{4}}{a_{4}}dz. \end{array}$$

The capacity of the **RD** channel is then given by:

(16) $$C_{r,FD}=\frac{2}{\lambda_{1}\lambda_{3}\lambda_{4}}\int_{0}^{\infty }\int_{0}^{\infty }e^{-\frac{z}{\lambda_{4}}}K_{0}\left(t_{4}\right)\frac{b_{4}z+c_{4}}{a_{4}}\log\left(1+\gamma \right)dzd\gamma .$$

Finally, the throughput $\tau_{FD}$ is given by:

(17) $$\tau_{FD}=\left(1-\alpha_{1}\right).\min\left\{C_{r,FD},C_{d,FD}\right\}.$$

 ☐

Table 1 below summarizes the analytical expressions of ergodic capacities and end-to-end throughput in HD and FD transmission modes.

## 3. Numerical Results and Discussion

### 3.1. Simulation Parameters

In our experiments, Matlab was used as the simulation tool because it contains efficient built-in functions to simplify the coding and accelerate the simulation speed. The geometrical settings of the model simulates a wireless V2V communication system in constrained environments such as car parks where transmitters and receivers are more static or slowly moving. We assume that the dedicated power station has an effective range of 10 m, or $d_{3}$ = 10 m. The distance between the power station and the destination is 30 m. The angle $\theta =135^{\circ }$, using geometry we can compute the **SD** distance (i.e., $d_{1}+d_{2}$) and maximum $d_{1}$ as 37.74 m and 16 m respectively. In the simulations, the distance $d_{1}$ is varied from 1 to the maximum $d_{1}$ with an increment of 1 m. We compute the $d_{2}$ and $d_{4}$ adaptively with each value of $d_{1}$ using geometry.

In the simulations, we compute the channel gains using the standard path loss model with carrier frequency $f_{c}$ = 2.4 GHz and path loss exponent *m* = 2.7. Moreover, we assume that the noise power per Hertz is −160 dBm, or −190 dB, giving the total noise power is 10${}^{-19}\times$ 100 kHz (transmitted bandwidth) = 10${}^{-14}$.

We assume that the energy harvesting circuitry at **S** and **R** ideally has maximum efficiency, η = 1. The transmitted power at the power station, *P* is set at 10 Watts and the SINR varies in a range [-*∞* −10 dB]. The mean values, $\lambda_{1}$, $\lambda_{2}$, $\lambda_{3}$ and $\lambda_{4}$ are all set to 1.

Our model considers imperfect SI cancellation in FD communication with the residual SI is proportional to the relay transmission power. The calculation of SINR is given by:

(24) $$\mathrm{SIN}R=\frac{\beta P_{r,FD}}{\mathrm{Total}\;\mathrm{noise}\;\mathrm{power}}=\frac{\beta P_{r,FD}}{10^{-14}}.$$

The energy efficiency $\eta_{EE}$ is defined as the number of bits transmitted by one Joules:

(25) $$\eta_{EE}=\frac{\tau }{P\alpha_{1}T+E_{CC}}$$

where $E_{CC}$ is the circuitry energy consumption and it is set at 1.5 W*T*.

### 3.2. Effects of Energy Harvesting Time

To investigate the effects of the energy harvesting time factor $\alpha_{1}$ on the throughput performance of HD and FD communications, we fix the relay location at $d_{1}$ = 9 m.

**Figure 4:**
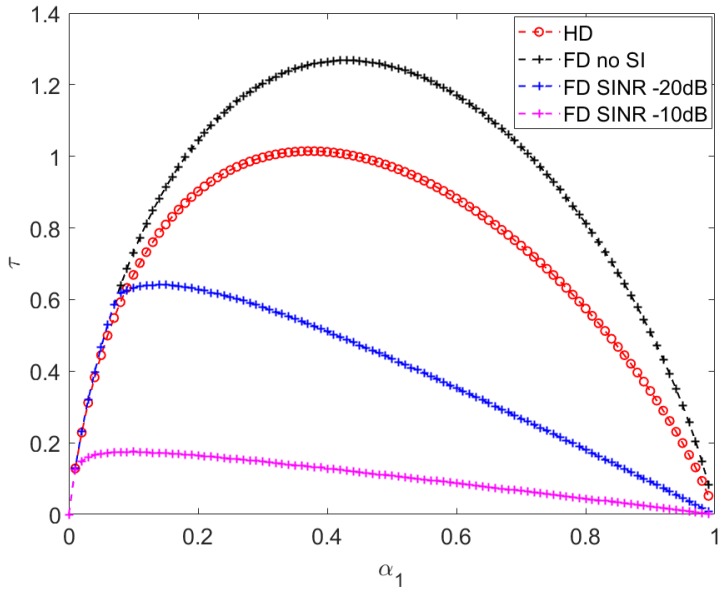
End-to-end throughput τ vs. harvesting time factor $\alpha_{1}$ with $d_{1}$ = 9, $d_{3}$ = 10, P=10.

Figure 4 shows that the throughput increases when $\alpha_{1}$ increases from 0 to an optimal value and starts decreasing beyond the optimal value of $\alpha_{1}$. This can be explained as smaller $\alpha_{1}$ values give less time for energy harvesting, causing less energy for transmission and a lower end-to-end throughput as a result. On the other hand, when $\alpha_{1}$ is beyond the optimal value, more time is spent for energy harvesting, but time for data transmission is constrained. Hence, it also leads to a lower throughput value.

Figure 4 also demonstrates that the optimal harvesting time increases when SINR decreases in FD mode. This is because lower SINR values impose slower increase of SI when the relay is harvesting more energy.

For a given $\alpha_{1}$ value, FD communication always has higher effective transmission time compared to HD. However, the transmission time advantage is not sufficient to compensate for the throughput loss caused by the SI trade-off. Therefore, in our simulations, HD mode only produces lower throughput than the no-SI FD communication as shown in Figure 4.

**Figure 5:**
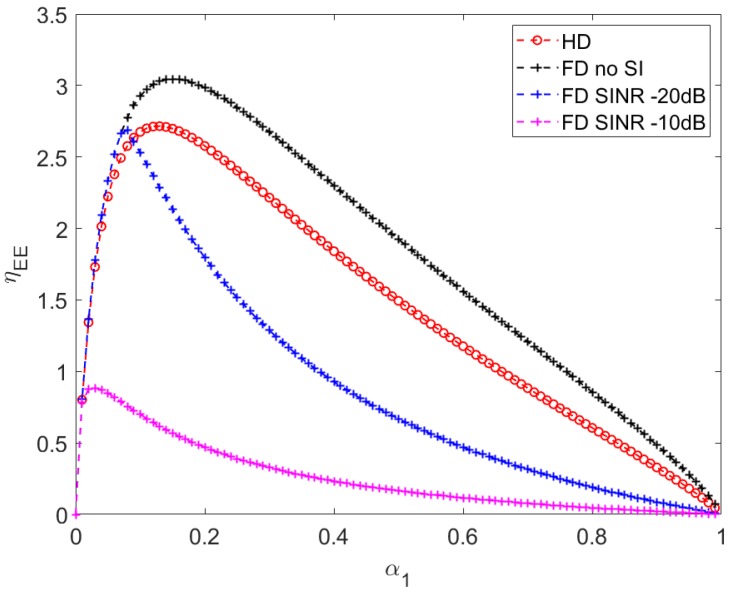
Energy efficiency $\eta_{EE}$ vs. harvesting time factor $\alpha_{1}$ with $d_{1}$ = 9, $d_{3}$ = 10, P=10.

Figure 5 reveals that energy efficiency increases when $\alpha_{1}$ increases from 0 to an optimal value and starts decreasing beyond the optimal $\alpha_{1}$ value. At smaller $\alpha_{1}$ values, the harvested energy is insignificant compared to the $E_{CC}$, causing lower energy efficiencies. Although larger $\alpha_{1}$ (i.e., beyond the optimal value) generate more harvested energy, it constrains the data transmission capacity and reduces the energy efficiency as a result.

### 3.3. Effects of Relay Location

In general, the maximum throughput improves when the relay moves from the source toward an optimal location and starts decreasing beyond that point. It can be observed that HD communication and FD communication with perfect SI cancellation outperform the others, particularly when $d_{1}$ is further from **S** (i.e > 50% **SR** length).

Additionally, HD and no-SI FD communication have optimal relay locations within the range of 6–10 m (±10% from the middle point of **SR**). The distance from the relay to the power station is shortest when the relay is at the middle of **SR**. Around this point, more harvested energy favors HD and no-SI FD communication.

In contrast, higher SINR FD communication (i.e., ≥−20 dB) has optimal relay locations closer to the source. After the end-to-end throughput achieves the peak, it plunges rapidly before it marginally decreases to a stable value. When $d_{1}$ increases from 1 to an optimal value, the relay harvests more energy for transmission, improving the end-to-end throughput. In this stage, the end-to-end throughput increase is driven by the capacity increase on the **RD** channel.

When SINR level is high, SI is quickly amplified as $d_{1}$ moves from the optimal locations to the middle point of **SR** due to the larger energy harvested by the relay, hence rapidly reduces the throughput on the **SR** channel. Beyond the middle point of **SR**, larger source-relay distance reduces the channel gain due to higher path loss, resulting in lower channel capacity on the **SR** link. Beyond the optimal relay location, the declination of the end-to-end throughput is dictated by the capacity reduction on the **SR** link.

**Figure 6:**
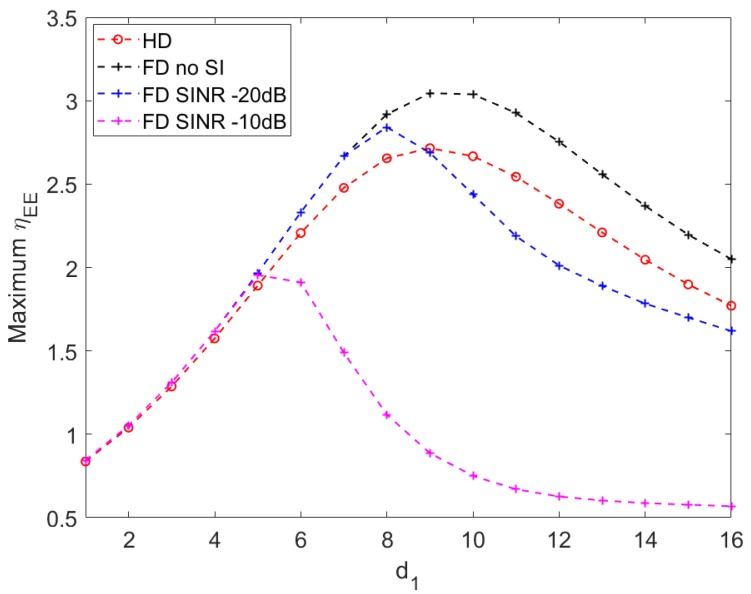
Maximum energy efficiency vs. distance from source to relay $d_{1}$ with $d_{3}$ = 10, P=10.

**Figure 7:**
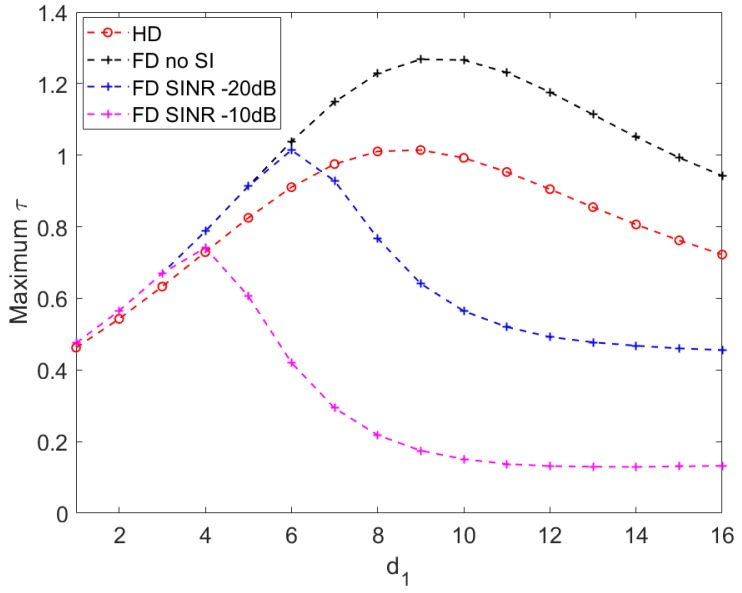
Maximum throughput vs. distance from source to relay $d_{1}$ with $d_{3}$ = 10, P=10.

Figure 6 suggests that in applications where energy efficiency is important (i.e., green communication), HD and no-SI FD communication offer highest achievable energy efficiencies in the range 6–10 m of $d_{1}$. This is consistent with results achieved in Figure 7. It leads to a result that highest end-to-end throughput with highest energy efficiency can be achieved within ±10% range from the middle point of the **SR** distance, using HD communication or FD with perfect SI cancellation. Nevertheless, HD communication can be more suitable in particular applications that tolerate certain throughput and efficiency performance, because it requires less implementation complexity than FD communication.

## 4. Conclusions

In this paper, a buffer-aided DF relaying model for V2V communication was investigated, which consists of energy-constrained source and relay harvest RF energy from a dedicated power station for data processing and transmission. The numerical results compare the throughput performance and the energy efficiency between HD and FD communications under the effects of the energy harvesting time and the relay deployment locations. To compute the throughput, we derived ergodic capacity expressions which are summarized in Table 1. The work in this paper can be extended by applying resource allocation techniques [17] and investigating a constrained delay at the relay [20]. In the future, we will also investigate the combination of energy harvesting with more complicated non-orthogonal multiple access (NOMA) or multi-point NOMA cooperative relay with various relaying protocols, as an extension of this work [21].

## Figures and Tables

**Table 1:** Summarized analytical expressions of ergodic capacities and end-to-end throughput.

Half-Duplex (HD)	Full-Duplex (FD)
Ergodic Capacity of **SR** channel	
(18) $$C_{r,HD}=\int_{0}^{\infty }\frac{2b_{1}}{\lambda_{1}\lambda_{3}}K_{0}\left(t_{1}\right)\log_{2}\left(1+\gamma \right)d\gamma$$ where, $t_{1}=\sqrt{\frac{4b_{1}\gamma }{\lambda_{1}\lambda_{3}}},\;b_{1}=\frac{\alpha_{2}\sigma_{sr}^{2}}{\eta P\alpha_{1}F^{2}d_{1}^{-m}d_{2}^{-m}}$	(19) $$C_{r,FD}=\frac{2}{\lambda_{1}\lambda_{3}\lambda_{4}}\int_{0}^{\infty }\int_{0}^{\infty }e^{-z/\lambda_{4}}K_{0}\left(t_{4}\right)\frac{b_{4}z+c_{4}}{a_{4}}\log_{2}\left(1+\gamma \right)dzd\gamma$$ where, $t_{4}=\sqrt{\frac{4\gamma (b_{4}z+c_{4})}{a_{4}\lambda_{1}\lambda_{3}}},\;a_{4}=\eta P\alpha_{1}F^{2}d_{1}^{-m}d_{3}^{-m},b_{4}=\eta P\beta \alpha_{1}Fd_{4}^{-m},\;c_{4}=\left(1-\alpha_{1}\right)\sigma_{sr}^{2}$
Ergodic Capacity of **RD** channel	
(20) $$C_{d,HD}=\int_{0}^{\infty }\frac{2b_{2}}{\lambda_{2}\lambda_{4}}K_{0}\left(t_{2}\right)\log_{2}\left(1+\gamma \right)d\gamma$$ where, $t_{2}=\sqrt{\frac{4b_{2}\gamma }{\lambda_{2}\lambda_{4}}},\;b_{2}=\frac{\alpha_{3}\sigma_{rd}^{2}}{\eta P\alpha_{1}F^{2}d_{2}^{-m}d_{4}^{-m}}$	(21) $$C_{d,FD}=\int_{0}^{\infty }\frac{2b_{3}}{\lambda_{2}\lambda_{4}}K_{0}\left(t_{3}\right)\log_{2}\left(1+\gamma \right)d\gamma F$$ where, $t_{3}=\sqrt{\frac{4b_{3}\gamma }{\lambda_{2}\lambda_{4}}}\;b_{3}=\frac{\left(1-\alpha_{1}\right)\sigma_{rd}^{2}}{\eta P\alpha_{1}F^{2}d_{2}^{-m}d_{4}^{-m}}$
End-to-end throughput	
(22) $\tau_{HD}=\min\left\{\alpha_{2}C_{r,HD},\alpha_{3}C_{d,HD}\right\}$	(23) $\tau_{FD}=\left(1-\alpha_{1}\right).\min\left\{C_{r,FD},C_{d,FD}\right\}$

## Appendix A

This appendix presents the working details to derive the CDF $F_{\gamma_{r,HD}}\left(\gamma \right)$ of $\gamma_{r,HD}$ in HD communication.

$$\begin{array}{cc} F_{\gamma_{r,HD}}\left(\gamma \right) & =\mathrm{Pr}(\gamma_{r,HD}<\gamma )\\ \; & =\mathrm{Pr}\left(\frac{\eta P\alpha_{1}|h_{1}{|}^{2}{\left|h_{3}\right|}^{2}}{\alpha_{2}\sigma_{sr}^{2}}<\gamma \right)\\ \; & =\mathrm{Pr}\left(e_{1}<\frac{b_{1}\gamma }{e_{3}}\right)\;\mathrm{where}\;b_{1}=\frac{\alpha_{2}\sigma_{sr}^{2}}{\eta P\alpha_{1}F^{2}d_{1}^{-m}d_{3}^{-m}}\\ \; & =\int_{0}^{\infty }f_{e_{3}}\left(x\right)\left(1-e^{-\frac{b_{1}\gamma }{\lambda_{1}x}}\right)dx\\ \; & =\int_{0}^{\infty }f_{e_{3}}\left(x\right)dx-\frac{1}{\lambda_{3}}\int_{0}^{\infty }e^{-\frac{x}{\lambda_{3}}-\frac{b_{1}\gamma }{\lambda_{1}x}}dx\\ \; & =1-t_{1}K_{1}\left(t_{1}\right) \end{array}$$

where $t_{1}=\sqrt{\frac{4b_{1}\gamma }{\lambda_{1}\lambda_{3}}}$ and $K_{1}\left(.\right)$ is the first order of modified Bessel function of the second kind, using (3.324.1, 518) [19].

## Appendix B

This appendix presents the working details to derive the CDF, $F_{\gamma_{r,FD}}$, of $\gamma_{r,FD}$ in FD communication.

(A1) $$\begin{array}{cc} F_{\gamma_{r,FD}}\left(\gamma \right) & =\mathrm{Pr}(\gamma_{r,FD}<\gamma )\\ \; & =\mathrm{Pr}\left(\frac{\eta P\alpha_{1}|h_{1}{|}^{2}{\left|h_{3}\right|}^{2}}{\eta P\beta \alpha_{1}{\left|h_{4}\right|}^{2}+\left(1-\alpha_{1}\right)\sigma_{sr}^{2}}<\gamma \right)\\ \; & =\mathrm{Pr}\left(\frac{a_{4}e_{1}e_{3}}{b_{4}e_{4}+c_{4}}<\gamma \right) \end{array}$$

where, $a_{4}=\eta P\alpha_{1}F^{2}d_{1}^{-m}d_{3}^{-m},b_{4}=\eta P\beta \alpha_{1}Fd_{4}^{-m},c_{4}=\left(1-\alpha_{1}\right)\sigma_{sr}^{2}$.

$F_{\gamma_{r,FD}}$ is then given by:

(A2) $$\begin{array}{cc} F_{\gamma_{r,FD}}\left(\gamma \right) & =\mathrm{Pr}\left(e_{1}.e_{3}<\frac{\gamma (b_{4}e_{4}+c_{4})}{a_{4}}\right)\\ \; & =\int_{0}^{\infty }f_{e_{4}}\left(z\right)F_{e_{1}.e_{3}}\left(\frac{\gamma b_{4}}{a_{4}}z+\frac{\gamma c_{4}}{a_{4}}\right)dz\\ \; & =\int_{0}^{\infty }\int_{0}^{\infty }\int_{0}^{\omega }f_{e_{4}}\left(z\right)f_{e_{1}}\left(x\right)f_{e_{3}}\left(y\right)dydxdz \end{array}$$

where $\omega =\frac{\gamma (b_{4}z+c_{4})}{a_{4}x}$ (using the product distribution rule). Because $e_{1}$, $e_{3}$ and $e_{4}$ are exponential random variables, $F_{\gamma_{r,FD}}$ is then given by:

(A3) $$\begin{array}{cc} F_{\gamma_{r,FD}}\left(\gamma \right) & =\frac{1}{\lambda_{1}\lambda_{3}\lambda_{4}}\int_{0}^{\infty }\int_{0}^{\infty }\int_{0}^{\omega }e^{-\frac{z}{\lambda_{4}}}e^{-\frac{x}{\lambda_{1}}}e^{-\frac{y}{\lambda_{3}}}dydxdz\\ \; & =-\frac{1}{\lambda_{1}\lambda_{4}}\int_{0}^{\infty }\int_{0}^{\infty }e^{-\frac{z}{\lambda_{4}}}e^{-\frac{x}{\lambda_{1}}}\left[e^{-\frac{\gamma (b_{4}z+c_{4})}{\lambda_{3}a_{4}x}}-1\right]dxdz\\ \; & =-\frac{1}{\lambda_{4}}\int_{0}^{\infty }e^{-\frac{z}{\lambda_{4}}}\left[t_{4}K_{1}\left(t_{4}\right)-1\right]dz\\ \; & =1-\frac{1}{\lambda_{4}}\int_{0}^{\infty }e^{-\frac{z}{\lambda_{4}}}t_{4}K_{1}\left(t_{4}\right)dz \end{array}$$

where $t_{4}=\sqrt{\frac{4\gamma (b_{4}z+c_{4})}{a_{4}\lambda_{1}\lambda_{3}}}$, using (3.324.1, 518) [19].
